# Lack of Evidence for Molecular Mimicry in HIV-Infected Subjects

**DOI:** 10.1371/journal.pone.0127662

**Published:** 2015-11-23

**Authors:** Peter D. Burbelo, James S. Klimavicz, Steve G. Deeks, Joseph A. Kovacs, Jack A. Ragheb

**Affiliations:** 1 Dental Clinical Research Core, National Institute of Dental and Craniofacial Research, National Institutes of Health, Bethesda, MD, United States of America; 2 Department of Medicine, University of California, San Francisco, San Francisco, CA, United States of America; 3 NIH Clinical Center, National Institutes of Health, Bethesda, MD, United States of America; 4 Office of Biological Products, OPQ, CDER, FDA, Silver Spring, MD, United States of America; University of Cape Town, SOUTH AFRICA

## Abstract

Previous studies in HIV patients have reported autoantibodies to several human proteins, including erythropoietin (EPO), interferon-α (IFN-α), interleukin-2 (IL-2), and HLA-DR, as potential mediators of anemia or immunosuppression. The etiology of these autoantibodies has been attributed to molecular mimicry between HIV epitopes and self-proteins. Here, the Luciferase Immunoprecipitation System (LIPS) was used to investigate the presence of such autoantibodies in HIV-infected adults. High levels of antibodies to HIV proteins such as capsid (p24), matrix (p17), envelope (gp41), and reverse transcriptase (RT) were detected using LIPS in both untreated and anti-retroviral-treated HIV-infected individuals but not in uninfected controls. LIPS readily detected anti-EPO autoantibodies in serum samples from subjects with presumptive pure red cell aplasia but not in any of the samples from HIV-infected or uninfected individuals. Similarly, subjects with HIV lacked autoantibodies to IFN-α, IL-2, HLA-DR and the immunoglobulin lambda light chain; all purported targets of molecular mimicry. While molecular mimicry between pathogen proteins and self-proteins is a commonly proposed mechanism for autoantibody production, the findings presented here indicate such a process is not common in HIV disease.

## Introduction

The exact mechanism(s) responsible for cell-mediated and humoral immune responses against self-proteins in different autoimmune diseases and other disorders often remains unknown. In some cases, it has been shown that autoantibodies are directed against modified proteins, mutant proteins or overexpressed proteins [1]. Molecular mimicry is another proposed etiology of autoantibody production. In this case, peptide sequence homology between a pathogen and a host protein gives rise to autoreactive T cells and/or antibodies that cross react with structurally similar host proteins, thus triggering autoimmunity [2]. However, in most cases of reported human molecular mimicry, the presence of cross reactive antibody responses between pathogen and cellular proteins has not been substantiated and the mechanism remains controversial [3].

During HIV infection, high levels of virus replication cause destruction of CD4+ T-cells, leading to a profound blunting of the immune system. Nonetheless, HIV infection induces high levels of antibodies against viral proteins including the HIV capsid, matrix and envelope proteins [4]. During long-term antiretroviral therapy (ART), plasma HIV RNA levels typically become undetectable, but antibodies directed against HIV proteins persist indefinitely [5,6]. Autoantibodies to cellular proteins that share short regions of sequence homology with HIV proteins have also been reported. These autoantibodies include those targeted against HLA-DR [7], interferon-α (IFN-α) [8], interleukin-2 (IL-2) [9], immunoglobulins [10] and EPO [11–13]. The presence of these antibodies has been inferred to cause immune suppression and anemia [7–9,11,12,14,15] In particular, anti-EPO autoantibodies have been found in 20% to 41% of untreated HIV patients and are associated with impaired erythropoiesis and HIV-associated anemia [11–13]. Despite these and other studies, the seroprevalence of autoantibodies against such cellular targets in HIV disease has not been substantiated employing defined recombinant proteins with new antibody profiling technologies.

Fluid-phase immunoassays are generally the most sensitive and specific method for identifying autoantibody responses against both conformational and linear epitopes [16,17]. The Luciferase Immunoprecipitation System (LIPS) is a fluid-phase immunoassay employing defined recombinant proteins that permits detection of antibodies against a variety of infectious and autoimmune targets [17]. Previous HIV studies with LIPS measured antibodies against the entire viral proteome [18] and identified unique antibody profiles in elite controllers and weak humoral responses in the Berlin patient, the only person cured of HIV [5,19]. In patients with HIV-associated immune reconstitution inflammatory syndrome, LIPS detected increased autoantibodies against several autoimmune-associated proteins [20]. In the current study, LIPS was used to investigate whether previously reported molecular mimicry-induced autoantibody responses against cellular proteins were present in a cohort of untreated and ART-treated HIV-infected adults.

## Methods

### Ethics statement

All subjects provided written informed consent. The studies were approved by the committee on Human Research, the Institutional Review Board of the University of California, San Francisco, UCSF, NIH Institutional Review Board and FDA Institutional Review Board.

### Study participants

Subject serum samples were collected from uninfected blood donors enrolled in studies at the NIH Clinical Center or from HIV patients enrolled in the SCOPE study at University of California, San Francisco [6]. The cohort included uninfected blood donors (n = 8), untreated HIV-infected subjects (n = 60), and ART-treated HIV subjects (n = 27). Ten of these 27 treated individuals had samples before and after at least four years of ART. From the seventy-seven different HIV-infected individuals, 88% (68/77) were male, 10% (8/77) were female and one was intersex. The median age was 45.2 years and the racial composition was 49% white, 26% African-American, 13% mixed, 5% Latino, 5% Asian, and 1% Pacific Islander. All treated HIV-infected subjects received ART for at least one year and had HIV RNA levels below detection limits using standard assays.

### Measurement of antibodies against HIV proteins by LIPS

Anti-HIV antibodies were measured by LIPS using previously described *Renilla* luciferase-HIV antigen constructs for p24, p17, gp41, and RT [18]. Antibody measurements by LIPS were performed at room temperature with a master plate of serum samples [21]. For antibody data analysis, raw light units (LU) were used.

### Detection of proposed mimicry-induced autoantibodies against cellular target proteins

LIPS was used to detect autoantibodies that have been proposed to arise due to molecular mimicry between HIV proteins and five cellular proteins (**Table 1**). Short HIV peptide sequences homologous to cellular proteins have been proposed as the mimicry triggers for autoantibody production (**Table 1**). Previously described *Renilla* luciferase C-terminal fusion proteins against EPO, IFN-α and IL-2 (exclusive of the signal peptide) were used to detect autoantibodies in this study [5,22]. In addition, two new *Renilla* luciferase C-terminal fusion proteins for HLA-DR and immunoglobulin lambda light chain (λ-LC) were generated. The primer adapter sequences used to clone each protein are as follows: for HLA-DR, 5′-GAGGG ATCCGGGGACACCCGACCACGT-3′ and 5′-GAGCTCGAGTCAGCTCAGGAATCCTGT-3′, and for λ-LC, 5′-GAGAGATCTTCCTATGAGCTGACACAG-3′ and 5′-GAGCTCGAGCTATGAACATTCTGTAGG-3′. Both DNA fragments encoding the proteins were then subcloned downstream of *Renilla* luciferase into the *BamH1-XhoI* site using the pREN2 vector and DNA sequencing was used to confirm their integrity.

**Table 1:** Proposed cellular targets of molecular mimicry in HIV subjects.

Cellular protein	Cellular peptide	HIV protein	HIV peptide	Reference
EPO	**L**I**C**D**SR**V**LER**	p17	**L**V**C**A**SR**E**LER**	[11–13]
IFN-α	**ILAV**KK**Y**FRRIT**L**	gp41	**ILAV**ER**Y**LKDQQ**L**	[8]
IL-2	**LE**RI**LL**	gp120	**LE**HL**LL**	[9]
HLA-DR	N**GT**E**RV**R	gp41	E**GT**D**RV**I	[7]
λ-LC	**GVETTTPS**	p24	**GVETTTPS**	[10]

Autoantibodies to these cellular proteins were tested by LIPS as described above for HIV proteins. Positive control serum or commercial antibodies were used to confirm each of the antibody tests for the five human proteins. The positive controls for the LIPS anti-EPO antibody assay consisted of a reference panel of monoclonal antibodies and clinical serum samples directed against EPO that were obtained through the National Institute for Biological Standards and Control, Potters Bar, England [23]. A serum sample from a previously identified anti-IFN-α seropositive thymoma patient was used as a positive control for that assay [22]. Although only a single anti-INF-α positive control sample was used for standardization in the current study, this LIPS assay has successfully detected INF-α autoantibodies in other known autoimmune cohorts, including thymoma [22] and lupus patients [24]. For the other targets, commercial monoclonal antibodies obtained from Santa Cruz Biotechnologies against IL-2 (sc-52017), HLA-DR (sc-51617), and λ-LC (sc-69923) were used to validate the corresponding LIPS antibody tests.

### Statistical analysis

GraphPad Prism 6 software (San Diego, CA) was used for statistical analyses. Median antibody levels are reported for each group. The non-parametric Mann-Whitney *U* test was used for comparison of antibody levels in the different subject groups. Tests for differences between HIV patients and controls with four HIV proteins and five autoantigens used a Bonferroni adjusted *P* value (*P* = 0.01) for significance. Only statistically significant values are shown in the Figs. The cut-off values for determining seropositivity against the cellular targets was based on the mean plus five standard deviations of a small number of healthy control samples.

## Results

### Study population demographics

To investigate the prevalence of autoantibodies against cellular proteins that may have arisen due to molecular mimicry, a cohort of 60 untreated HIV subjects, 27 ART-treated HIV subjects and 8 uninfected blood donors were studied. The untreated HIV subjects had a median viral load of 36,548 copies RNA/ml [Interquartile range (IRQ): 17,694–85,370]. The median CD4+ T cell count in the untreated-HIV subjects was 376 cells/mm^3^ (IQR: 259–506) and 684 cells/mm^3^ (IQR: 443–872) in the ART-treated HIV subjects.

**Fig 1:**
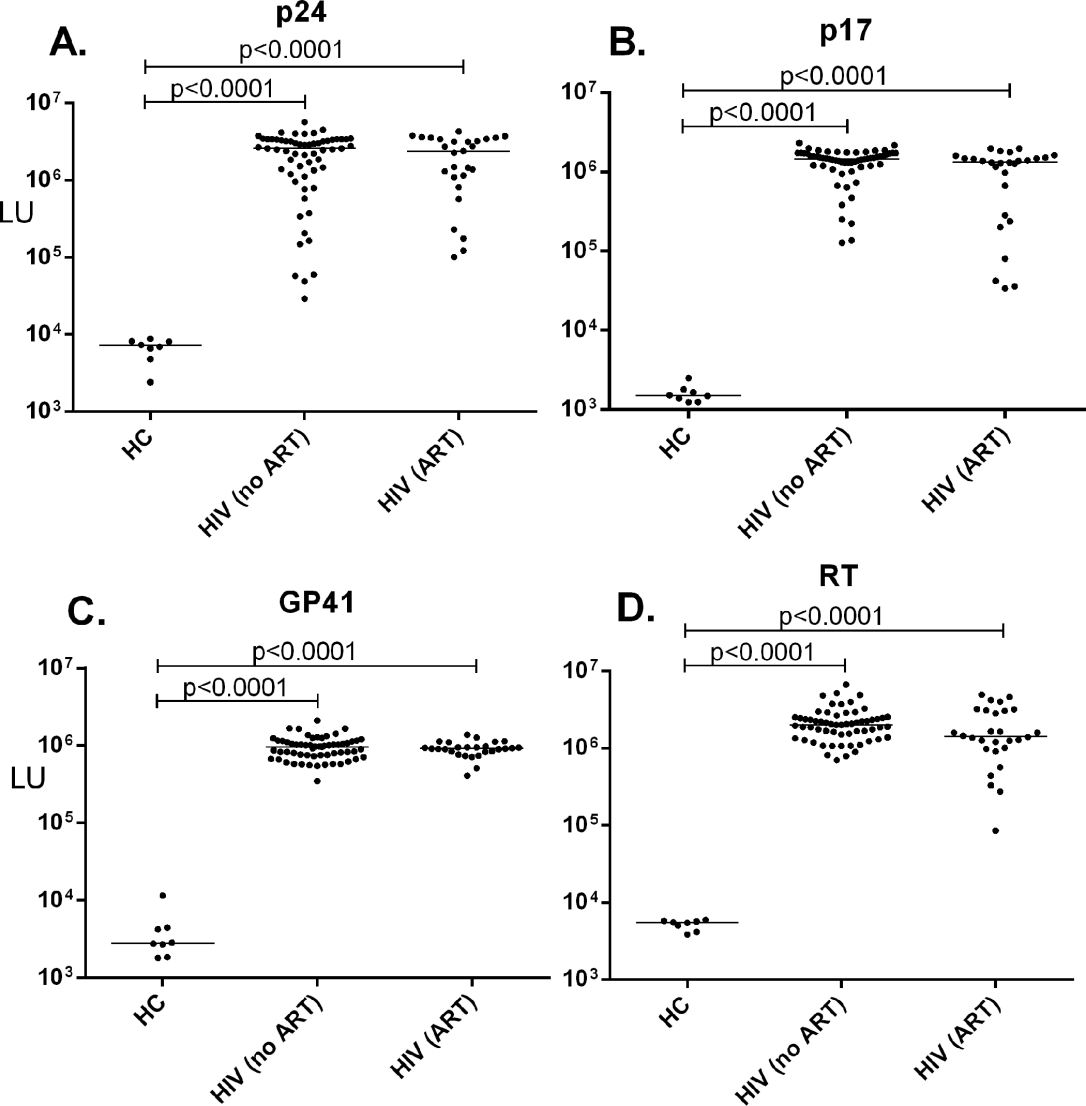
Antibody responses to HIV proteins. LIPS was used to detect antibodies against four HIV proteins in eight healthy controls (HC), HIV-infected subjects before ART (n = 60), and after ART (n = 27). The level of antibody (in LU) against the HIV proteins p24 (A), p17 (B), gp41 (C), and RT (D) is plotted on the y-axis using a log_10_ scale. Each symbol in the graph represents one sample from one subject. The horizontal line represents the median LU value in each group. Statistically significant differences were calculated with the Mann-Whitney *U* test and only statistically significant values are shown.

The level of serum antibodies against four HIV proteins (p24, p17, gp41, and RT) was measured using LIPS. Compared to uninfected controls, high levels of anti-p24 antibodies were detected in HIV-infected individuals before and after ART (**Fig 1A**). Similarly, immunoreactivity against p17, gp41 and RT was 100 to 1000-fold higher in HIV subjects compared to healthy controls (**Fig 1B–1D**). Consistent with our previous study [5], there were statistically significant differences (p<0.0001) between the HIV groups and uninfected blood donor controls, but there were no differences in the antibody levels against these four HIV proteins before and after ART-induced virologic control (**Fig 1A–1D**).

### No evidence of anti-EPO autoantibodies in HIV subjects

**Fig 2:**
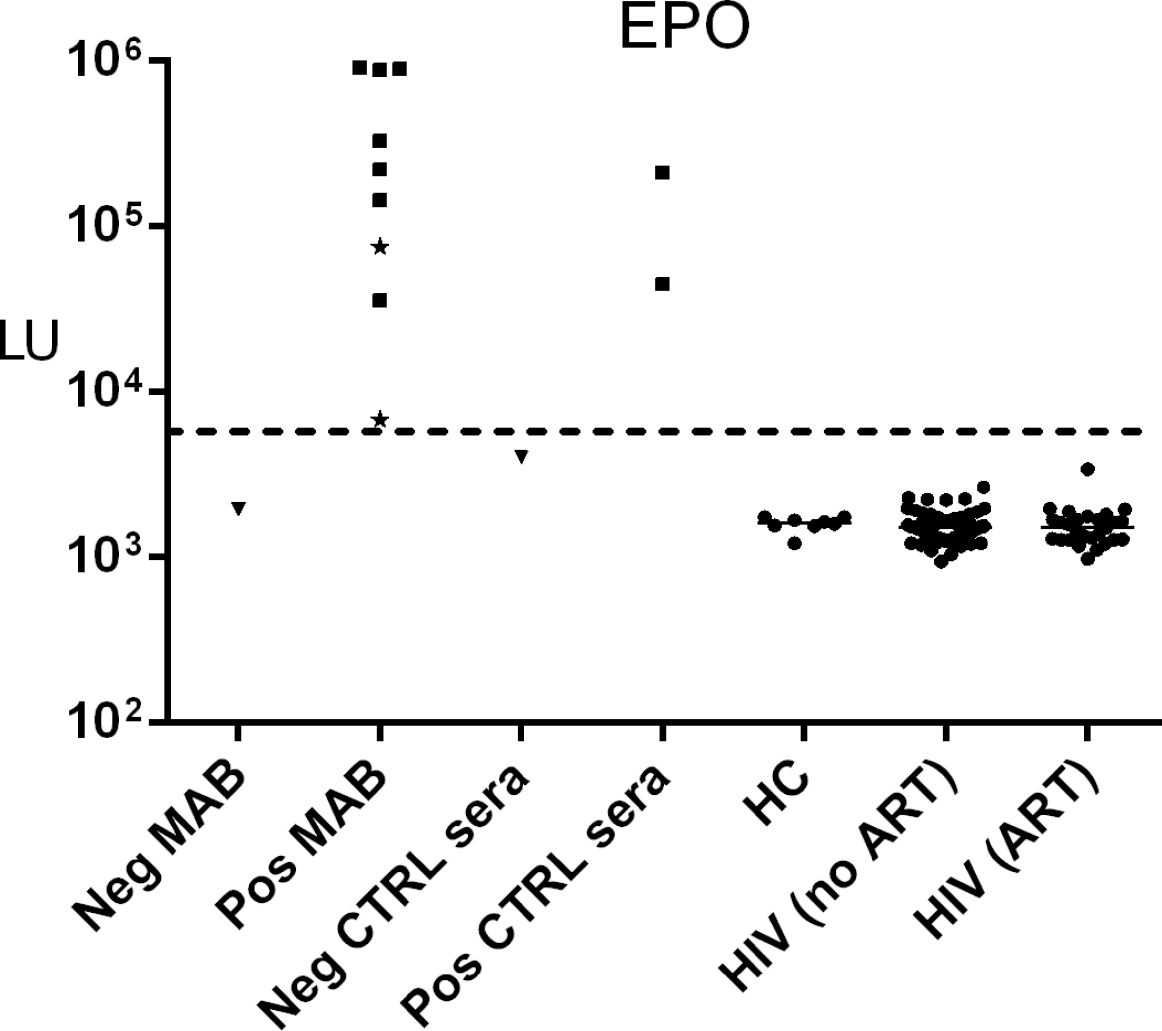
No evidence of anti-EPO autoantibodies in HIV patients. Negative (Neg) and positive (Pos) control (CTRL) monoclonal antibodies (MAB) to EPO, as well as anti-EPO Neg and Pos CTRL clinical sera were assayed using LIPS. Two of the anti-EPO monoclonal antibodies were of the IgM isotype (marked by stars), which is an immunoglobulin isotype that binds poorly to protein A/G beads. Anti-EPO antibody levels were also measured in eight healthy controls (HC), HIV patients before ART (n = 60), and after 3–7 years of ART (n = 27). The anti-EPO antibody levels in LU are plotted on the y-axis using a log_10_ scale. The median anti-EPO antibody level in the healthy controls and HIV patient groups are shown by the horizontal line. The dotted line represents the cut-off value for determining seropositivity for EPO.

A high frequency of anti-EPO antibodies, presumably driven by molecular mimicry of a similar peptide in the HIV p17 protein, has been reported in HIV disease [11–13]. In order to independently confirm the presence of anti-EPO antibodies in HIV subjects using LIPS, the assay was first validated using known positive and negative anti-EPO monoclonal antibodies and clinical samples from a panel that was part of a World Health Organization collaborative study to evaluate reference human monoclonal antibodies against EPO [23]. Using LIPS, all the anti-EPO monoclonal antibodies and seropositive clinical samples scored positive, while all of the negative control monoclonal antibodies and seronegative clinical samples scored negative (**Fig 2**). The positive control monoclonal anti-EPO antibodies had LIPS values between 6,793 and 917,900 LU. Similarly, the two positive clinical samples had anti-EPO antibody levels of 44,911 and 212,892 LU. When linear peptides derived from predicted target epitopes [13] in the p17 protein of HIV (LVCASRELERFAVNPGLLE) and EPO (APPRLICDSRVLERYLLEAK) were used in LIPS as competitors (up to 30 μg/ml), they failed to inhibit binding of known anti-EPO antibody samples to their target. Similarly, they did not inhibit binding of HIV seropositive samples to the p17 target, indicating that both these antibodies recognize conformational rather than linear epitopes (data not shown). As shown in (**Fig 2**), anti-EPO autoantibodies were undetectable in HIV positive and negative serum samples with all samples having background LU values. No statistical difference in the anti-EPO LIPS signal was found between the control blood donors and HIV subjects, demonstrating that there are no anti-EPO autoantibodies in our HIV subjects. Lastly, in agreement with the lack of shared B-cell epitopes between p17 and EPO, testing of anti-EPO monoclonal and human clinical samples in the p17-LIPS assay failed to demonstrate any immunoreactivity (**S1 File**).

### Autoantibodies against IFN-α, IL-2, HLA-DR and λ-LC are absent in HIV subjects

**Fig 3:**
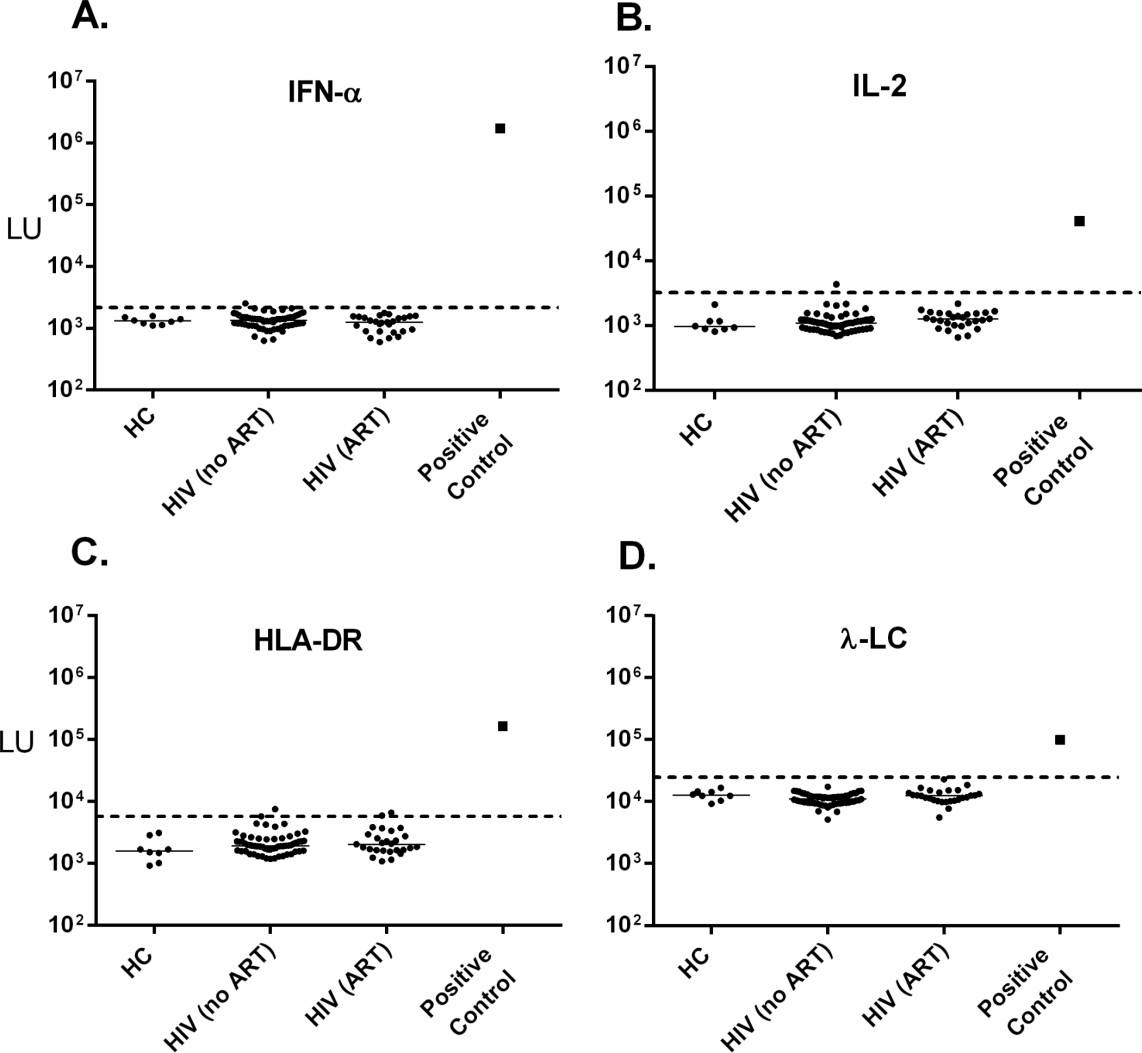
Lack of autoantibodies against IFN-α, IL-2, HLA-DR, and λ-LC in HIV-infected subjects. Autoantibody levels in LU as determined by LIPS are shown for (A) IFN-α, (B) IL-2, (C) HLA-DR, and (D) λ-LC on the y-axis using a log_10_ scale. The LU value for each of the positive control antibodies (closed squares) used to validate the assays is shown in its respective plot. The dotted line represents the cut-off value for determining seropositivity.

The HIV cohort was also screened for autoantibodies against several other proposed targets of molecular mimicry, namely IFN-α, IL-2, HLA-DR, and λ-LC (**Table 1**). As described in the material and methods, a positive control serum or monoclonal antibodies were used to validate the LIPS autoantibody test for each of the four proteins. As expected, a positive control serum sample from a previously described patient with thymoma-related opportunistic infections showed highly elevated levels of anti-IFN-α autoantibodies (**Fig 3A**). However, testing of serum samples from controls and HIV subjects demonstrated no autoantibodies to IFN-α with LU values below the cut-off, which were similar to the buffer control (**Fig 3A**).

Since there are no established human autoimmune diseases containing autoantibodies against IL-2, MHC-DR or λ-LC, commercial antibodies, which produced robust signals in LIPS, were employed as positive controls (**Fig 3B–3D**). In contrast to the positive controls, there was no statistical difference in the LIPS signal between the control blood donors and HIV subjects in any of the four autoantibody tests for these proposed protein targets (**Fig 3B–3D**). Overall, the immunoreactivity observed in the HIV-infected subjects against these four targets was at background levels for these assays. A few subjects showed antibodies values just above the cut-offs, but these antibodies are unlikely to be physiologically relevant because they were 10- to 1000-fold lower than the positive controls and likely reflect non-specific binding.

## Discussion

In this study we attempted to reproduce the previously reported findings [7–9,11–13,25] of autoantibodies that have been proposed to cause anemia or contribute to immunodeficiency in HIV subjects. In contrast to those reports, our results demonstrate that there are no autoantibodies against the five, proposed cellular targets of molecular mimicry in HIV subjects. Immunoreactivity against these proteins in HIV patient sera is indistinguishable from that of healthy blood donors and buffer blanks. Many of the seventy-seven untreated HIV patients had low CD4 counts associated with immunosuppression, thus making them an appropriate cohort for studying the potential role of autoantibodies against IL-2, IFN-α, HLA-DR and λ-LC, in the pathogenesis of HIV-induced immunosuppression. Our investigation incorporated a number of important advances not present in earlier studies. First, we employed the LIPS fluid-phase immunoassay, rather than solid phase immunoassays, to study antibodies against viral and cellular proteins thought to be involved in molecular mimicry. Using LIPS, HIV subjects demonstrated 100- to 1,000-fold higher immunoreactivity against HIV viral proteins compared to healthy controls. Second, positive control clinical serum samples and other antibodies were used to confirm the capacity of LIPS to detect autoantibodies against the five cellular targets. In each case, the positive controls produced signals at least 10- to 100-fold higher than that observed in HIV subjects or healthy blood donors. For each of the five cellular proteins, no statistically significant difference in autoantibodies was detected between the HIV subjects and the healthy controls.

We postulate that the autoantibodies found in HIV subjects during previous studies were likely false positive signals in the earlier immunoassays. Western blotting and ELISA was used in several of these studies to detect autoantibodies against endogenous proteins from cell extracts including IL-2 [9], IFN-α [8] and HLA-DR [7]. Western blotting is known to suffer from poor specificity due to the false assignment of immunoreactivity to a molecular weight species. None of the previous autoantibody studies in HIV subjects used appropriate positive control antibodies to validate the performance of their immunoassays or performed functional testing to show whether the autoantibodies they detected were neutralizing. In addition, one often cited example of molecular mimicry, the GKKSTS peptide shared between HIV gp41 and the lambda light chain immunoglobulin, is taken from a review article [10] published without any corresponding experimental immunoassay evidence. Although it is conceivable that there are autoantibodies only detectable by the solid phase immunoassays against some of the targets, their clinical relevance has not been proven and would require additional studies to show their existence and functional significance.

It is important to point out that the erythropoietin ELISA originally used to detect anti-EPO autoantibodies in SLE [26] and then in HIV patients [11,12] has not been rigorously validated and is known to be prone to high rates of false positive results [27,28]. In the three studies reporting anti-EPO autoantibodies in HIV associated with anemia, well-characterized anti-EPO antibody samples from patients with pure red cell aplasia were not used for validation, but rather SLE and HIV patient sera was employed [11–13]. Despite our inability to demonstrate any anti-EPO seropositive HIV subjects by LIPS, our study was well-powered to detect the 23.5% and 41% prevalence of anti-EPO autoantibodies reported in untreated HIV-infected subjects [11,12]. The absence of anti-EPO autoantibodies by LIPS in HIV subjects and other cohorts is consistent with the idea that these antibodies are rare and occur most commonly after administration of recombinant EPO therapy [21,22,27–29]. If antibodies against p17 did drive the generation of anti-EPO autoantibodies and anemia in HIV patients, it is difficult to reconcile the reported decrease in EPO autoantibodies following one year of ART [12] because highly elevated levels of anti-p17 and other HIV antibodies persist and remain unchanged years after HAART [5,6]. Thus a simple model of molecular mimicry does not explain how anti-EPO autoantibodies would decrease following HAART and reverse anemia when the triggering anti-p17 antibodies remain unchanged. One plausible explanation is that p17 antibodies with host antigen cross-reactivity are under more stringent tolerance control and are eliminated while p17 antibodies that are not cross-reactive persist. However, based on the reported strong correlation between increased CD4 and red blood cell counts in HIV-treated subjects [12], a more likely explanation is that HAART underlies the improvement in the immunological and hematological parameters of these patients.

Many other short sequences in human proteins with homology to HIV have been identified using bioinformatics [30]. One central assumption of the HIV molecular mimicry hypothesis is that the shared peptide sequences are sufficient to drive antibody production against cellular proteins. Our finding that the immunoglobulin light chain sequence GVETTTPS, which is identical to a peptide in HIV gp41, is not a target of autoantibodies underscores the observation that not all short HIV peptides are antigenic. In this and other cases in which HIV and cellular proteins share amino acid sequences, functional B cell tolerance may be maintained because of spatial masking of the sequences, the presence of non-homologous substitutions that disrupt important linear and/or conformational epitopes, lack of adequate T cell help, or active regulatory processes that prevent an antibody response. Overall, our findings provide important evidence against a role for molecular mimicry in driving autoantibody production and contributing to HIV- associated pathogenesis.

## Supporting Information

S1 File No evidence of immunoreactivity of anti-EPO monoclonal and human anti-EPO sera with HIV p17. Seven different IgG monoclonal antibodies (EPO MAB) against EPO, one Neg Ctrl MAB, two anti-EPO seropositive clinical sera (EPO Pos), one anti-EPO seronegative human sera (EPO Neg), two HIV subjects (HIV) and a buffer blank were assayed for immunoreactivity against HIV p17 by LIPS. The anti-p17 antibody levels in LU are plotted on the y-axis using a log_10_ scale. Only the HIV patients demonstrated anti-p17 immunoreactivity.
(TIF)
